# Pituitary Macroadenoma and Adamantinomatous Craniopharyngioma: A Rare Case Report of Sellar Collision Tumors

**DOI:** 10.1155/crnm/6895334

**Published:** 2025-09-04

**Authors:** LaToya McLean, Carrie Andrews, Louis Cappelli, Grant Gillan, Mark Curtis, James J. Evans, Wenyin Shi

**Affiliations:** ^1^Department of Radiation Oncology, Thomas Jefferson University, Philadelphia, Pennsylvania 19107, USA; ^2^Department of Neurological Surgery, Thomas Jefferson University, Philadelphia, Pennsylvania 19107, USA; ^3^Department of Pathology, Anatomy & Cell Biology, Thomas Jefferson University, Philadelphia, Pennsylvania 19107, USA

**Keywords:** case report, collision tumors, craniopharyngioma, pituitary adenoma, radiation, sella tumors

## Abstract

We present a rare case of a collision tumor involving a pituitary macroadenoma and adamantinomatous craniopharyngioma in a 49-year-old woman. The patient presented with a 2-year history of amenorrhea and elevated prolactin. Brain MRI revealed two sellar masses. Initially managed with observation due to the absence of neurological deficits, surgical resection was later performed after clinical and radiographic progression. Pathology confirmed both tumor types: pituitary macroadenoma and adamantinomatous craniopharyngioma. Postoperative MRI showed residual disease at the superior margin. The patient subsequently received fractionated stereotactic radiation for residual disease and tolerated well.

## 1. Introduction

Collision tumors are an extremely rare phenomenon defined by the coexistence of two histologically distinct neoplasms in the same anatomical location. While collision tumors have been reported, comprehensive data on their overall incidence remain limited. A retrospective study of reported cases across all body sites found collision tumors in the sellar region to be particularly uncommon, occurring in only 11 of 2359 cases (0.47%) [1]. The most frequently observed combination involves pituitary adenoma and Rathke's cleft cyst. Less commonly, pituitary adenomas collide with craniopharyngiomas, with only 15 such cases documented in the literature [1–4]. Management typically involves surgical resection, though treatment can be challenging due to the rarity of the condition and the need for individualized approaches. This case report explores the embryogenesis, histopathology, clinical presentation, management strategies, and potential complications of a 49-year-old female with a sellar collision tumor of a pituitary macroadenoma and an adamantinomatous craniopharyngioma.

## 2. Case Presentation

**Figure 1:**
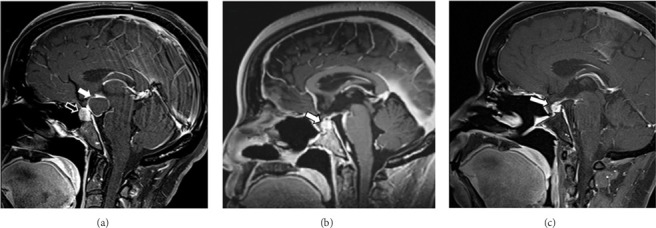
MRIs: (a) preoperative MRI: MRI postcontrast demonstrating a heterogeneously enhancing lesion seen in the left sellar measuring 1 cm × 9 mm × 1 cm (open arrow). There is a contiguous suprasellar mass which is predominantly cystic measuring 2.9 × 2.8 × 2.7 cm (solid white arrow). (b) Postoperative MRI showed a residual enhancing lesion in the sella and along the infundibulum (arrow). There is a decreased bulk of residual enhancing tissue in the sella compared to preoperative imaging. This demonstrates slightly decreased T1 hyperintense signal abnormality/enhancement in the right suprasellar region extending towards the interpeduncular cistern. (c) 6 months follow-up MRI showed the residual enhancing lesion in the sella decreased from the previous study (arrow).

A 49-year-old woman with a medical history of leiomyomas (with prior myomectomy), hypertension, anemia, and irritable bowel syndrome presented with a two-year history of amenorrhea. She denied galactorrhea or visual disturbances but reported longstanding memory impairment. Endocrinologic workup revealed elevated prolactin (49.8 ng/mL; normal range: 4.8–23.3 ng/mL) and abnormal cortisol levels both pre- and postdexamethasone suppression (24.9 and 1.4 μg/dL; normal range: 6.2–19.4 μg/dL). ACTH was within the expected range (36.8 pg/mL; normal range: 7.2–63.3 pg/mL), while FSH (25.4 mIU/mL; normal range: 3.5–134.8 mIU/mL depending on cycle phase) and estradiol (655.0 pg/mL; normal range < 6–498 pg/mL depending on cycle phase) were normal. Normal pituitary hormone levels indicated that the adenoma was nonfunctional. MRI of the sella revealed a 1.1 cm left pituitary macroadenoma and an abutting 2.2 cm posterior suprasellar mass with mass effect on the optic chiasm and mammillary bodies (Figure 1(a)). The case was reviewed at a multidisciplinary tumor board. Given minimal lab abnormalities and no visual changes or optic apparatus compression, the decision was made for a short-interval follow-up with repeat imaging and endocrine labs every 6 months, with intervention planned if tumor growth or clinical changes occurred.

After 1 year of surveillance, the patient presented to the emergency department with worsening symptoms of dizziness, brain fog, memory loss, and visual disturbances. MRI of the brain revealed an increase in the size of the suprasellar cystic component of the mass. The patient underwent successful endoscopic transnasal transsphenoidal resection of both sellar masses. The postoperative course was complicated by a thalamic infarct and adrenal insufficiency, requiring admission to the neurosurgical intensive care unit for close monitoring.

**Figure 2:**
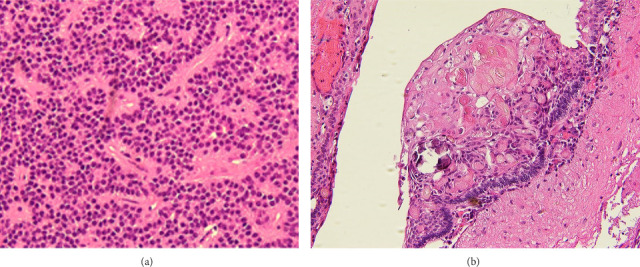
Pathology: (a) gonadotroph pituitary adenoma (H&E, A-20 ×, B-40 ×): bland, monotonous nuclei with minimal cytoplasm in a uniform, sheet-like distribution. These cells often characteristically form perivascular pseudorosette-like structures. Gonadotroph pituitary adenoma/PNET (A-GATA3, B-SF-1, C-FSH, D-LH, 20 ×): Lesional cells staining diffusely positive for GATA3 (A), SF-1 (B), FSH (C), and LH (D), together supporting a gonadotrophic origin. (b) Adamantinomatous craniopharyngioma (H&E, 20 ×): lesion with palisading squamous epithelium (arrow) and areas of “wet” keratin (arrowhead) that are histologically diagnostic of craniopharyngioma.

Intraoperatively, the tumor was found to be densely adherent to the bilateral mammillary bodies. After achieving decompression of the large anterior cranial base and suprasellar mass, further resection was halted to avoid injury to the mammillary bodies and adjacent critical neurovascular structures. Postoperative MRI 2 days after surgery confirmed that the majority of the mass identified on previous imaging had been resected; however, a small area of enhancement along the superior surgical margin was suspicious for residual disease. Further resection was not pursued to avoid damage to the mammillary bodies and other adjacent neurovascular structures. Pathological examination revealed both gonadotrophin-secreting pituitary adenoma and adamantinomatous craniopharyngioma. There was no significant mutation detected, including BRAF mutation (Figure 2).

Endocrine lab results, including prolactin, TSH, LH, and FSH, remained largely within normal limits, except for ACTH, which was low (< 9 pg/mL; normal range: 9–46 pg/mL). The patient's adrenal insufficiency was managed with glucocorticoid replacement. She continued to experience peripheral visual field deficits, diplopia, and fatigue, and was discharged to a rehabilitation facility. Follow-up MRIs showed stable disease, and the patient continued physical rehabilitation (Figure 1(b)). MRI 6 months postsurgery showed decreased bulk of residual enhancing tissue in the sella (Figure 1(c)). She was admitted to the hospital a few times due to syncope and was diagnosed with dehydration every time she was admitted. She continued to experience blurred vision and double vision, although both symptoms were gradually improving. At the radiation oncology evaluation, the role of radiation therapy (RT) for the residual tumor was carefully considered, especially given its proximity to the optic chiasm and nerves. Considering the tumor's location and the risk of neurological injury, a dose of 54 Gy in 30 fractions was recommended to maximize local control while minimizing toxicity. However, the decision was made to defer radiation and continue observation, allowing further recovery. MRI 1 year after surgery showed no significant change in the residual enhancing lesion in the sella and along the infundibulum. She remained on hydrocortisone 10 mg twice daily for adrenal insufficiency. She had stable visual deficits, and a decision was made to proceed with RT. The patient was treated using volumetric modulated arc therapy (VMAT) to a total dose of 54 Gy in 30 fractions. Radiation was delivered to the postoperative bed, encompassing the surgical defect and regions of residual enhancement. Treatment planning incorporated the fusion of the preoperative, postoperative, and most recent MRIs with the planning CT for accurate target and organ-at-risk delineation. Planning risk volumes (PRVs) were created for the brainstem, optic chiasm, and bilateral optic nerves. All dose constraints were met, with the highest doses delivered to the brainstem PRV and optic chiasm PRV, each receiving a maximum dose of approximately 55 Gy. She tolerated radiation treatment well. MRI 1 year after radiation showed stable findings. Endocrine laboratory values remained within normal limits at both 2 months and 1 year following RT, indicating preserved pituitary function. She will continue routine follow-up.

## 3. Discussion

The tumorigenesis of collision tumors remains incompletely understood, and the potential interactions between the tumors are still unclear. Collision tumors are rare, with only 16 reported cases of pituitary adenoma and craniopharyngioma combinations in the literature to date [5]. These tumors involve distinct histological types, each with unique genetic alterations, growth patterns, tumor microenvironments, and responses to treatment. In the case of pituitary adenoma and craniopharyngioma, it remains uncertain whether there is a synergistic or competitive relationship between the two. Craniopharyngiomas arise from persistent embryonic remnants of Rathke's pouch, which typically give rise to the adenohypophysis during embryogenesis. In contrast, pituitary adenomas originate from abnormal proliferation of pituitary cells within the adenohypophysis, without involvement of Rathke's pouch remnants. Pituitary adenomas are benign monoclonal neoplasms and can be classified based on their hormonal secretion profile and molecular characteristics. Molecular and immunohistochemical analysis is performed to demonstrate lineage-specific transcription factor expression (e.g., Pit-1, SF-1, and T-Pit) and mutation profiling consistent with pituitary adenoma subtypes as well as strong positivity for pituitary hormones (including GNAs in GH-secreting tumors and USP8 in corticotroph adenomas), confirming its monoclonal nature and distinguishing it from the craniopharyngioma component. The interaction between collision tumors in the sellar region is not well defined, though it is believed that the unique microenvironment of the region, such as local hormonal and immune responses, may play a role [1, 5, 6]. Understanding this complex interplay is crucial for predicting tumor behavior and developing targeted therapies.

The diagnosis of collision tumors in the sellar region is typically made following a high clinical suspicion, confirmed with MRI, which provides detailed information on the location, imaging characteristics, and relationship of the lesions with each other and surrounding structures. Initial management may involve either observation or surgical intervention, with the approach determined through multidisciplinary discussions involving surgeons, oncologists, and the patient. Surgical resection is the primary treatment for collision tumors; however, achieving gross total resection can be challenging due to the proximity of critical anatomical structures. This requires a careful balance between tumor debulking and preserving function to ensure maximal safe resection [7, 8]. If the patient is not a candidate for surgery, or if gross total resection is not achievable, RT may be considered for local control.

RT can be used to treat sellar lesions either in a neoadjuvant or adjuvant setting, depending on the treatment objectives [9]. In the neoadjuvant setting, RT is employed to reduce tumor size, slow tumor progression, or improve surgical outcomes by shrinking the tumor, thereby minimizing the risk of damage to adjacent structures during surgery. In the adjuvant setting, RT aims to eliminate microscopic residual disease, prevent local recurrence, and stabilize hormone function [10–12]. Salvage radiation refers to treatment administered after initial curative intent therapy (e.g., surgery) when recurrence or progression, as defined by RECIST criteria, occurs. Most studies and guidelines focus on the role and outcomes of postoperative radiotherapy. The National Comprehensive Cancer Network (NCCN) guidelines recommend adjuvant radiation as the standard of care for central nervous system tumors, including craniopharyngiomas, but do not address neoadjuvant RT [13].

One study comparing the outcomes of adjuvant radiotherapy plus surgery versus surgery alone for recurrent nonfunctioning pituitary adenomas found a significant improvement in progression-free survival (PFS) with adjuvant RT compared to surgery alone [8]. While no significant difference in PFS was observed between adjuvant and salvage RT, salvage RT demonstrated a better toxicity profile and should be considered over a second surgery in cases of recurrence or progression [14]. The specific dosing and fractionation regimens for RT in collision tumors of the sellar region would be based on general principles of radiation oncology, tailored to the individual patient's clinical scenario, including factors such as the extent of surgical resection, tumor histology, and proximity to critical structures [15, 16]. When considering postoperative radiation, it is crucial to evaluate whether adjuvant or salvage radiation is more appropriate. Although limited data exist comparing these approaches in the context of sellar collision tumors, the decision to administer postoperative radiation should be made through a multidisciplinary approach to optimize patient outcomes.

This case highlights the rare coexistence of a nonfunctional pituitary adenoma and craniopharyngioma, a rarer tumor combination compared to the more frequently reported prolactin-secreting adenomas. The tumor's dense adherence to critical neurovascular structures created significant surgical challenges, requiring subtotal resection followed by targeted image-guided radiation, a management approach less commonly detailed in previous reports that often describe gross total resection. In addition, the use of advanced multimodal MRI/CT fusion in VMAT planning and the preservation of endocrine function postradiation highlight important multidisciplinary considerations.

## References

[1] Gong L., Chen H., Zhang W. (2022). Primary Collision Tumors of the Sellar Region: Experience From a Single Center. *Journal of Clinical Neuroscience*.

[2] Bteich F., El Khoury L., Nohra G., Trak V., Yazbek S., Akiki M. (2020). Pituitary Adenoma and Papillary Craniopharyngioma: A Rare Case of Collision Tumor and Review of the Literature. *World Neurosurgery*.

[3] Zhao Y., Zhang H., Lian W. (2017). Collision Tumors Composed of Meningioma and Growth Hormone-Secreting Pituitary Adenoma in the Sellar Region: Case Reports and a Literature Review. *Medicine (Baltimore)*.

[4] Finzi G., Cerati M., Marando A. (2014). Mixed Pituitary Adenoma/Craniopharyngioma: Clinical, Morphological, Immunohistochemical and Ultrastructural Study of a Case, Review of the Literature, and Pathogenetic and Nosological Considerations. *Pituitary*.

[5] Snyder R., Fayed I., Dowlati E., Seager A., Mason R. B. (2019). Pituitary Adenoma and Craniopharyngioma Collision Tumor: Diagnostic, Treatment Considerations, and Review of the Literature. *World Neurosurgery*.

[6] Koutourousiou M., Kontogeorgos G., Wesseling P., Grotenhuis A. J., Seretis A. (2010). Collision Sellar Lesions: Experience With Eight Cases and Review of the Literature. *Pituitary*.

[7] Lucas J. W., Bodach M. E., Tumialan L. M. (2016). Congress of Neurological Surgeons Systematic Review and Evidence-based Guideline on Primary Management of Patients With Nonfunctioning Pituitary Adenomas. *Neurosurgery*.

[8] Charleux T., Vendrely V., Huchet A. (2022). Management After Initial Surgery of Nonfunctioning Pituitary Adenoma: Surveillance, Radiotherapy or Surgery?. *Radiation Oncology*.

[9] Minniti G., Jaffrain-Rea M. L., Osti M., Cantore G., Enrici R. M. (2007). Radiotherapy for Nonfunctioning Pituitary Adenomas: From Conventional to Modern Stereotactic Radiation Techniques. *Neurosurgical Review*.

[10] Chanson P., Raverot G., Castinetti F., Cortet-Rudelli C., Galland F., Salenave S. (2015). French Endocrinology Society Non-Functioning Pituitary Adenoma w-g: MBnagement of Clinically Non-Functioning Pituitary adenoma. *Annales d’Endocrinologie*.

[11] Fong K. Y., Lim M. J. R., Fu S. (2023). Postsurgical Outcomes of Nonfunctioning Pituitary Adenomas: A Patient-Level Meta-Analysis. *Pituitary*.

[12] Mantziaris G., Pikis S., Chytka T. (2023). Adjuvant Versus On-Progression Gamma Knife Radiosurgery for Residual Nonfunctioning Pituitary Adenomas: A Matched-Cohort Analysis. *Journal of Neurosurgery*.

[13] Horbinski C., Nabors L. B., Portnow J. (2023). NCCN Guidelines Insights: Central Nervous System Cancers, Version 2.2022: Featured Updates to the NCCN Guidelines. *Journal of the National Comprehensive Cancer Network*.

[14] Godil S. S., Tosi U., Gerges M. (2022). Long-Term Tumor Control After Endoscopic Endonasal Resection of Craniopharyngiomas: Comparison of Gross-Total Resection Versus Subtotal Resection With Radiation Therapy. *Journal of Neurosurgery*.

[15] Gupta T., Chatterjee A. (2020). Modern Radiation Therapy for Pituitary Adenoma: Review of Techniques and Outcomes. *Neurology India*.

[16] Scaringi C., Agolli L., Minniti G. (2018). Technical Advances in Radiation Therapy for Brain Tumors. *Anticancer Research*.

